# Shaping teachers' challenge appraisals of organizational transition: the roles of grassroots leadership, peer collaboration, and boundary-spanning experience

**DOI:** 10.3389/fpsyg.2026.1949606

**Published:** 2026-10-07

**Authors:** Haiying Jiang, Shuo Huang

**Affiliations:** 1 College of Architectural and Engineering, Guangzhou Polytechnic University, Guangzhou, China; 2 Guangdong University of Finance, School of National Finance, Guangzhou, China

**Keywords:** boundary-spanning experience, cognitive appraisal of transition, grassroots leadership, Job Demands-Resources (JD-R) model, moderated mediation, work psychology

## Abstract

**Introduction:**

Amidst the specialized vocational education reforms, primary-level teaching organizations are evolving into open and adaptive systems. Drawing upon the Job Demands-Resources (JD-R) model and Cognitive Appraisal Theory, this study examines the psychological mechanisms through which grassroots leadership, peer collaboration, and perceived organizational adaptation relate to teachers' positive cognitive appraisals of transition.

**Methods:**

A cross-sectional survey was conducted among 965 vocational college teachers. A moderated mediation model was utilized to test the sequential associations mapping from leadership and collaboration to teachers' cognitive appraisals of transition via organizational adaptation, alongside the potential moderating role of individual boundary-spanning experience (i.e., industry-academy double-qualified experience).

**Results:**

The path analysis reveals that grassroots leadership is positively associated with perceived organizational adaptation both directly and sequentially through teacher collaboration. These interrelated resources are concurrently linked to positive cognitive appraisals of transition. Furthermore, the hypothesized moderation effect of boundary-spanning expertise was not statistically supported, indicating that positive structural agility of the organization is universally associated with educators' cognitive appraisals across the cohort.

**Discussion:**

The study contributes to work psychology by investigating the concurrent interrelationships of environmental resources during systemic organizational change. It suggests that rather than individual boundary-spanning experience acting as a primary moderating factor, a highly adaptable organizational climate operates as a structurally supportive environment that relates to educators' positive cognitive adjustment.

## 1 Introduction

Driven by systemic policy reforms, vocational education is experiencing a paradigm shift that demands moving away from rigid hierarchical management toward open, collaborative, and agile organizational structures (Fidanboy et al., 2021; Sporn and Godonoga, 2024). Initiatives such as industry-education integration, digital curriculum overhauls, and the cultivation of dual-credentialed (“double-qualified”) educators have thrust primary-level teaching organizations into a highly volatile internal and external matrix. Consequently, these foundational instructional units—once strictly tasked with executing administrative directives—have evolved into decentralized hubs responsible for curriculum innovation, professional development, and navigating complex educational reforms. From a work stress perspective, this rapid expansion of responsibilities acts as an environmental demand, constantly challenging teachers' traditional routines and requiring significant cognitive and emotional adjustment.

Functioning as the immediate psychosocial work environment for educators, these localized organizations serve as the critical nexus between macro-institutional strategies and micro-level instructional execution. Viewed through the lens of the Job Demands-Resources (JD-R) model (Bakker and Demerouti, 2007; Demerouti et al., 2001), while macro-level reforms impose psychological demands on teachers, localized organizational attributes act as crucial job resources. Effectively mobilizing these relational and structural resources is essential for buffering educators' work stress, enhancing role clarity, and cultivating a climate of psychological safety within the vocational institution (Groulx et al., 2024; Kurt et al., 2024).

To systematically explain how teachers adapt to this turbulent environment, we integrate the JD-R model with Lazarus and Folkman's (1984) Cognitive Appraisal Theory of Stress. According to this integrated framework, when macro-level disruptions cascade down to the instructional level, their psychological impact is determined by how teachers cognitively appraise these environmental changes. We conceptualize teachers' positive cognitive appraisals of transition not merely as a passive description of structural features, but as their active, challenge-oriented cognitive appraisal of the transition. Under this appraisal process, grassroots supportive leadership and teacher collaboration function as relational job resources, whereas perceived organizational adaptation serves as a proximal environmental cue indicating that the teaching unit is actively adjusting to shield its members from reform uncertainty.

While a substantial body of literature has explored vocational education reforms, team building, and structural management, a critical theoretical lacuna remains. First, the predominant focus has heavily leaned toward objective structural adjustments and policy implementation, often neglecting the subjective psychological dimensions—specifically, how frontline educators cognitively appraise these ongoing changes. Second, the underlying pathways and boundary conditions remain a black box; we lack empirical evidence explaining how relation-oriented resources (leadership and collaboration) concurrently associate with positive appraisals through the pathway of overall organizational adaptation. Finally, existing studies tend to treat teachers as a homogeneous group, overlooking the role of individual coping resources. It remains unexplored whether teachers' practical backgrounds (such as “double-qualified” status or industry experiences), acting as personal resources, buffer the impact of change and moderate the paths related to the cognitive appraisal process (Oreg, 2003).

To address these gaps, the present study deployed a quantitative survey among 965 vocational college faculty members to test a moderated mediation model. Specifically, this study addresses the following four interconnected research questions:

RQ1: What is the baseline profile of teachers' appraisals of transition, grassroots leadership, peer collaboration, and perceived organizational adaptation in vocational colleges?

RQ2: Do grassroots leadership, peer collaboration, and perceived organizational adaptation significantly covary with teachers' positive cognitive appraisals of transition?

RQ3: Is there a theoretically aligned sequential association mapped from relational job resources (leadership and collaboration) to teachers' cognitive appraisals through perceived organizational adaptation?

RQ4: Does individual boundary-spanning experience (i.e., double-qualified or industry experience) moderate the relationship between perceived organizational adaptation and teachers' cognitive appraisals of transition?

This study contributes to work psychology by clarifying the conditional pathways through which environmental resources relate to teachers' transition appraisals. By analyzing these concurrent associations, the current research offers an integrated perspective on how structural adaptation and individual boundary-spanning experience interface to support teachers' cognitive adjustment during periods of systemic reform (Zyberaj et al., 2022).

## 2 Theoretical foundations and hypotheses

### 2.1 Ecological transformation of primary-level teaching organizations in vocational colleges

In this study, primary-level teaching organizations refer to the foundational functional units within vocational colleges that directly execute instructional delivery, pedagogical research, curriculum development, and talent cultivation. These encompass various configurations, including traditional teaching and research sections, agile course groups, and industry-education integration teams.

“Ecological transformation” in this context transcends mere structural reorganization; it denotes a systemic, adaptive evolution of the reciprocal relationships among these instructional units, their internal resources, and a turbulent external environment driven by policy shifts, industrial demands, and digital disruptions (Sudhartio et al., 2023).

Crucially, this study captures the cognitive appraisals of transition as the subjective, cognitive appraisal of transition (Lazarus and Folkman, 1984) by faculty and localized administrators regarding their unit's environmental sensitivity, resource permeability, digital integration, and symbiotic vitality. This construct centers on the organizational members' psychological representation and challenge-oriented interpretation of how open, connected, and resilient their primary-level unit is.

To establish theoretical clarity, this study strictly delineates the boundaries between the subjective cognitive appraisals of transition and perceived organizational adaptation. While both involve subjective evaluations, perceived organizational adaptability describes the unit's concrete operational agility (acting as a proximal environmental cue—e.g., the observable act of modifying a syllabus or shifting workloads). In contrast, the cognitive appraisal of transition captures the teacher's internal psychological and emotional response to these macro-level changes (acting as the psychological outcome—e.g., viewing the overall ecosystem's shift as a positive, developmental opportunity rather than a threat). Drawing upon the Cognitive Appraisal Theory of Stress (Lazarus and Folkman, 1984), the Job Demands-Resources framework (Demerouti et al., 2001), and Dynamic Capabilities Theory (Teece et al., 1997), perceived organizational adaptability is conceptualized here as teachers' perceived adaptation behaviors—representing their subjective cognitive representation of the active, operational adjustments the unit executes to mitigate external pressures. These perceived adaptive behaviors function as proximal environmental cues. When a unit is perceived to demonstrate agility, it relates to reduced ambiguity, which is associated with positive cognitive appraisals of transition. Under a cross-sectional design, perceived organizational adaptation and cognitive appraisals of transition are expected to operate in a synchronous, reciprocal covariance within the educators' daily work environment.

### 2.2 Primary-level teaching organization leadership

Primary-level teaching organization leadership encompasses the specific behaviors of localized leaders—such as teaching section directors, specialty construct leaders, and integration team heads—whose roles center on providing directional guidance, supportive empowerment, and resource coordination.

Unlike top-tier institutional executives, grassroots instructional leaders occupy a highly contextualized psychosocial nexus. They mediate between macro-strategic mandates and the micro-realities of daily teaching (Ghamrawi et al., 2024). Consequently, their leadership requires a nuanced blend of sensemaking and emotional support to orchestrate task division while fostering a collaborative climate.

Aligning with classical frameworks of Transformational Leadership (Carless et al., 2000) and Empowering Leadership (Arnold et al., 2000), this study contextualizes localized leadership through four distinct dimensions: (1) directional guidance, helping teachers cognitively deconstruct overwhelming macro-reforms into manageable tasks; (2) democratic empowerment, actively respecting members' pedagogical autonomy; (3) psychological safety for exploration, cultivating an error-tolerant climate for digital and industrial experimentation; and (4) resource buffering.

Viewed through the lens of the JD-R model, grassroots leadership acts as an essential relational job resource. By fostering psychological safety and reducing resource scarcity, supportive leadership is expected to relate to lower change anxiety, which is associated with positive cognitive appraisals of the unit's transition (Lin and Mohammad, 2025; Schmitz et al., 2025). Therefore, the following hypothesis is proposed:

H1: Primary-level teaching organization leadership is positively associated with teachers' positive appraisals of transition.

### 2.3 Teacher collaboration

Teacher collaboration transcends superficial collegiality, encompassing deep-level interdependent behaviors aimed at co-designing curricula, jointly addressing student developmental needs, and engaging in continuous pedagogical reflection (De Jong et al., 2022; Wijarwadi et al., 2025; Yilmaz, 2022).

Systemic adaptation is rarely sustained by isolated individuals; it relies on collective synergy. Psychologically, teacher collaboration facilitates the formation of Shared Mental Models (SMMs). When educators dismantle traditional occupational silos to collaboratively interpret curriculum standards, this interpersonal exchange is associated with collective efficacy (De Weerdt et al., 2024; Wullschleger et al., 2025). The crystallization of SMMs relates to lower psychological isolation, linking the organizational environment to perceptions of openness, adaptation, and transition. Concurrently, grassroots leaders displaying transformational and empowering behaviors are expected to provide the necessary institutional scaffolding for this collaboration to thrive. Based on this rationale, the following hypotheses are proposed:

H2: Teacher collaboration is positively associated with teachers' positive appraisals of transition.

H3: Primary-level teaching organization leadership is positively associated with teacher collaboration.

### 2.4 Perceived organizational adaptation

Perceived organizational adaptation denotes the perceived adaptive behaviors and continuous improvement exhibited by primary-level units in reconfiguring task deployments, updating curriculum contents, and realigning school-enterprise workflows in response to shifting policy demands and technological disruptions (Hjort et al., 2021; Hlatjwako et al., 2024). In contrast to the cognitive appraisal of transition (a subjective cognitive outcome), adaptation emphasizes teachers' perceptions of these operational adjustments and behavioral agility executed by the instructional unit (Talaja and Golem, 2024).

Conceptually anchored in Denison and Mishra's (1995) models of organizational culture and Holt et al.'s (2007) readiness for change paradigms, adaptation at the grassroots vocational level manifests practically: overriding rigid utilitarian boundaries to agilely reset work plans, recalibrating syllabi to match emerging industrial technologies, and sustaining systemic resilience by unblocking learning channels for educators.

At the operational level, a highly adaptable organization generates positive environmental cues that relate to lower ambiguity. Under the Cognitive Appraisal framework (Lazarus and Folkman, 1984), these concrete structural actions are associated with reduced change anxiety, subsequently relating to positive cognitive appraisals of transition (Burdziej et al., 2024; Kurt et al., 2024). Furthermore, this structural agility co-occurs with shared mental models among peers (collaboration) and democratic empowerment from local administrators (leadership), which are associated with minimized internal resistance (Goddard et al., 2007; Vangrieken et al., 2015). Under a cross-sectional design, these perceived adaptive behaviors are hypothesized to relate positively to other core variables as follows:

H4: Perceived organizational adaptation is positively associated with teachers' positive appraisals of transition.

H5: Teacher collaboration is positively associated with perceived organizational adaptation.

H6: Primary-level teaching organization leadership is positively associated with perceived organizational adaptation.

Integrating these pathways, the Job Demands-Resources framework suggests that relational job resources (leadership and collaboration) do not operate in isolation. Rather, supportive leadership and peer collaboration are expected to relate to structural agility (organizational adaptation), which in turn relates to positive cognitive appraisals of transition. Therefore, we propose a sequential mediation pathway consistent with theoretical expectations:

H7: Primary-level teaching organization leadership is indirectly and theoretically associated with teachers' positive appraisals of transition through the serial path of teacher collaboration and perceived organizational adaptation.

### 2.5 The moderating role of boundary-spanning resources

According to Cognitive Appraisal Theory (Lazarus and Folkman, 1984), individuals do not evaluate environmental demands identically; rather, their appraisals are conditioned by personal coping resources. In vocational settings, teachers with certified “double-qualified” status or rich industry experience possess distinct dual-identities. These teachers operate as boundary spanners between academia and industry. We conceptualize this experience as an individual boundary-spanning resource. In practice, these distinct professional categories (no designated experience, rich industry practice, and formal double-qualified certification) reflect a progressive accumulation of industry-academia exposure. Consequently, this study operationalizes this conceptual variable as an ordinal continuum representing increasing levels of external embeddedness, positing that such resources relate to higher career adaptability (Zyberaj et al., 2022).

During organizational transitions (such as syllabus restructuring or school-enterprise integration), teachers are exposed to substantial work adjustment. For teachers with high boundary-spanning resources, their familiarity with industry practices allows them to decode these adaptive changes as opportunities and challenges. In contrast, teachers lacking these external resources are more likely to perceive identical organizational adaptations as threats to their established pedagogical routines (Oreg, 2003). Although a highly adaptive organization provides fundamental structural support for all its members, we expect that teachers equipped with external industry resources are positioned to leverage this agility more effectively. Therefore, we propose that boundary-spanning resources act as a contextual moderator that conditions the link between organizational adaptation and transition appraisals:

H8: Individual boundary-spanning resources (i.e., certified double-qualified status or industry experience) moderate the positive association between perceived organizational adaptation and teachers' positive appraisals of transition, such that this association is stronger for teachers with higher boundary-spanning resources.

Synthesizing the theoretical framework and Hypotheses H1 through H8 developed above, the conceptual relationships among these core variables investigated in this study are illustrated in Figure 1.

**Figure 1:**
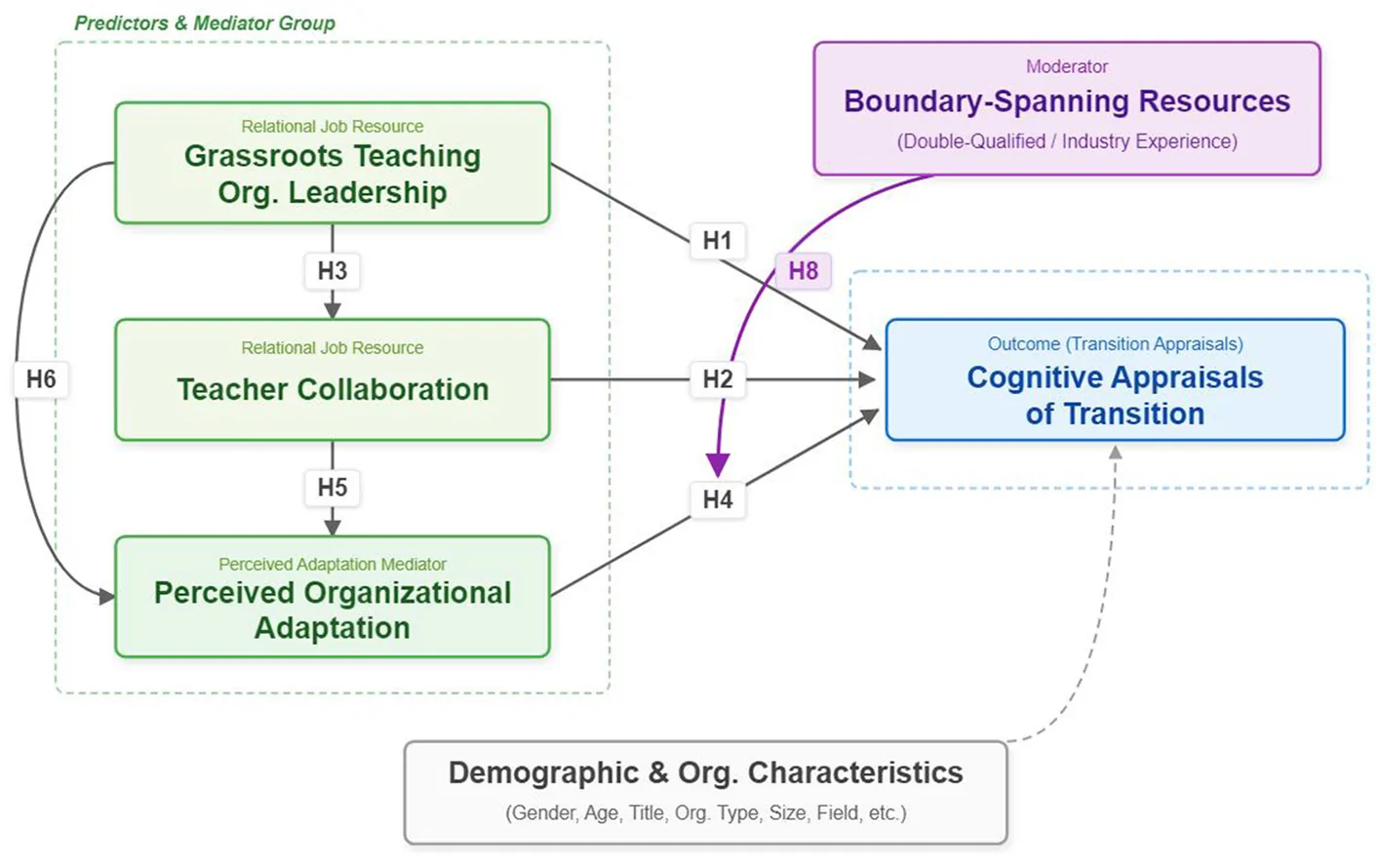
Hypothesized moderated mediation model.

## 3 Research design and methods

### 3.1 Research design

This study utilized a quantitative, cross-sectional self-report design to test the proposed moderated mediation model encompassing relational job resources (leadership and collaboration), structural adaptation, and educators' cognitive appraisals of transition. This approach is configured for delineating the macro-level profiles of these variables, validating the latent structure of the measurement instruments, and testing the statistical covariances among the constructs. In alignment with the cross-sectional nature of the data, the analysis focuses on diagnosing synchronous covariation, concurrent psychological states, and associative patterns within the organizational environment, without asserting causal directionality.

### 3.2 Data sources, quality control, and sample characteristics

#### 3.2.1 Target population and ethical protocols

The target demographic comprised full-time and part-time faculty and instructional administrators (aged 18 and above) actively engaged in core academic duties—such as teaching, curriculum design, and pedagogical research—within vocational colleges. To ensure ecological validity, eligibility required participants to be intimately familiar with the daily operational dynamics of at least one primary-level teaching unit (e.g., a teaching section or course group).

Data were collected via an online survey matrix systematically distributed through faculty networks, academic forums, and professional development cohorts using a non-probability convenience and snowball sampling approach. While efforts were made to distribute links across diverse geographical regions and institutions, exact institutional response rates could not be calculated due to the anonymous digital distribution method. To prevent duplicate submissions, respondent IP addresses and device identifiers were cross-checked within the survey platform. Furthermore, specific institutional or organizational-unit identifiers were deliberately not collected to ensure strict respondent anonymity; hence, the data nested structure precluded multilevel modeling. Strict adherence to ethical standards for human subjects was maintained. A mandatory digital informed consent module was positioned at the entry point of the survey. The survey design embedded spatial block-page segregation and strategically interspersed attention-check items to filter out unengaged responding. Participants retained the right to withdraw from the study at any point.

#### 3.2.2 Data screening and demographic profile

During the initial deployment, 1,000 raw responses were harvested. To ensure the integrity of the dataset, stringent exclusion criteria were applied post-collection. Cases were systematically eliminated based on lack of consent, ineligibility, failure of attention checks, excessive missing data (missing >3 core items), or unengaged responding patterns (e.g., straight-lining). This rigorous attrition process yielded a final, highly robust sample of 965 valid cases, reflecting a retention rate of 96.5% (see Table 1).

**Table 1:** Data cleaning conditions.

Data cleaning item	Quantity	Proportion of raw sample
Raw questionnaires	1,000	100.0%
Informed consent not provided	8	0.8%
Ineligible to participate	7	0.7%
Attention check failed	12	1.2%
Missing more than 3 core items	5	0.5%
Obvious straight-line responding	3	0.3%
Invalidated by any rule	35	3.5%
Final valid questionnaires	965	96.5%

Following data cleaning, descriptive frequency analyses were executed on 11 demographic and occupational covariates. The comprehensive demographic profile of the 965 educators is detailed in Table 2.

**Table 2:** Basic characteristics of the sample.

Variable	Category	Quantity	Percentage
Gender	Female	485	50.3%
	Male	453	46.9%
Other/Prefer not to say	27	2.8%
Age	18–30 years old	145	15.0%
	31–40 years old	367	38.0%
	41–50 years old	300	31.1%
	51 years old and above	139	14.4%
	Prefer not to answer	14	1.5%
Highest degree	Junior college and below	29	3.0%
	Bachelor's degree	225	23.3%
	Master's degree	634	65.7%
	Doctoral degree	77	8.0%
Professional title	Unrated	70	7.3%
	Primary (Junior)	204	21.1%
	Intermediate	434	45.0%
	Associate senior	215	22.3%
	Senior	42	4.4%
Years of working in vocational colleges	3 years and below	160	16.6%
	4–10 years	345	35.8%
	11–20 years	331	34.3%
	21 years and above	129	13.4%
Years of working in current primary-level teaching organization	Less than 1 year	102	10.6%
	1–3 years	279	28.9%
	4–6 years	216	22.4%
	7–10 years	208	21.6%
	More than 10 years	160	16.6%
Current primary identity	Full-time teacher	544	56.4%
	Part-time teacher	69	7.2%
	Teaching-research section director/Course group leader	165	17.1%
	Specialty leader/Professional group leader	97	10.1%
	School-level teaching management personnel	55	5.7%
	School/College-level (secondary) teaching management personnel	20	2.1%
	Other	15	1.6%
Primary-level teaching organization type	Teaching and research section	321	33.3%
	Professional teaching team	186	19.3%
	Course group	166	17.2%
	Professional group teaching organization	151	15.6%
	Teaching innovation team	76	7.9%
	Industry-education integration teaching team	45	4.7%
	Other	20	2.1%
Organizational scale	5 people and below	144	14.9%
	6–10 people	331	34.3%
	11–20 people	303	31.4%
	21–30 people	130	13.5%
	31 people and above	57	5.9%
Specialty or course field	Electronic Information	154	16.0%
	Finance and Trade	133	13.8%
	Equipment Manufacturing	124	12.8%
	Education and Sports	107	11.1%
	Medicine and Health	100	10.4%
	Culture and Art	75	7.8%
	Transportation	66	6.8%
	Civil Engineering and Architecture	65	6.7%
	Tourism	63	6.5%
	Agriculture, Forestry, Animal Husbandry, and Fishery	38	3.9%
	Public Administration and Service	27	2.8%
	Other	13	1.3%
“Double-qualified” teacher status or industry practical experience	Yes, certified as a “double-qualified” teacher	465	48.2%
	Yes, possessing rich industry practical experience	241	25.0%
	No	215	22.3%
	Uncertain	44	4.6%

As depicted in Table 2, the sample exhibits substantial heterogeneity across gender, age brackets, educational attainment, professional tenure, and institutional typologies. Notably, the cohort is anchored predominantly by mid-career professionals (aged 31–50) holding master's degrees and intermediate-to-senior titles. While population-level benchmarking data is not available to verify exact representativeness, the broad demographic distribution provides a diverse sub-sample for subsequent statistical modeling, though external validity should be interpreted with appropriate caution.

### 3.3 Psychometric instruments

This study operationalized the “Questionnaire on Ecological Transformation of Primary-level Teaching Organizations in Vocational Colleges.” The core inventory comprises four validated subscales encompassing 44 items, uniformly rated on a 5-point Likert-type scale (1 = strongly disagree, 5 = strongly agree). Higher aggregate scores reflect stronger endogenous perceptions or higher organizational manifestations of the specific construct. Reverse-coded items underwent standard score inversion prior to matrix analysis. Complete instruments and backend coding dictionaries are detailed in Supplementary materials S1 and S2.

#### 3.3.1 Cognitive appraisals of transition

This 12-item scale assesses teachers' cognitive appraisals of their unit's environmental sensitivity, resource permeability, organizational agility, and symbiotic vitality. While historically grounded in organizational ecology (Hannan and Freeman, 1977), this study conceptualizes these items psychologically as measuring teachers' challenge-oriented appraisals of the ecological transformation (Lazarus and Folkman, 1984). Specifically, the items are conceptually structured to capture the respondents' internal psychological and emotional evaluation of the shifting environment, rather than serving as an objective audit of operational agility. A sample item is: “Our organization rapidly recalibrates to shifts in vocational policies and industrial demands.” Two items (ET6, ET12) were reverse-coded.

#### 3.3.2 Primary-level teaching organization leadership

Comprising 10 items, this scale gauges localized administrators' behaviors concerning directional sensemaking, professional empowerment, and resource buffering functioning here as a measure of relational job resources. Rooted in the Global Transformational Leadership (Carless et al., 2000) and Empowering Leadership (Arnold et al., 2000) scales, it was contextually refined. A sample item is: “Our leader provides the necessary scaffolding and resources for pedagogical innovation.” Items GL7 and GL10 were reverse-coded.

#### 3.3.3 Teacher collaboration

Measured via 12 items, this variable captures the depth of collective pedagogical practices, cooperative curriculum design, and peer socio-emotional support within the JD-R relational framework. Driven by established professional collaboration inventories (Goddard et al., 2007; Vangrieken et al., 2015), a sample item reads: “Educators in our unit collaboratively co-construct course modules and practical projects.” Items TC5 and TC8 were reverse-coded.

#### 3.3.4 Perceived organizational adaptation

This 10-item scale evaluates the teachers' perceptions of organizational adaptive behaviors and operational agility of the instructional unit in restructuring workflows and curricula amidst external disruptions. It serves as the proxy for proximal environmental structural cues. Anchored in Denison and Mishra's (1995) cultural adaptability criteria and Holt et al.'s (2007) change readiness matrix, a sample metric is: “Our unit promptly reconfigures work deployments in response to evolving educational mandates.” Items OA4 and OA10 were reverse-coded.

#### 3.3.5 Background variables and grouping variables

To control for confounding variables and facilitate sub-group analyses, the survey captured structural attributes including gender, age, degree, tenure, localized identity, organizational schema, and discipline. Crucially, teachers' certified “double-qualified” (dual-credential) status or rich industry practical experience was captured via categorical items. This specific variable serves as the operational proxy for individuals' “boundary-spanning experience” in the moderation model. Within the context of vocational education, acquiring “double-qualified” certification is a cumulative professional milestone that strictly requires prior validated industry experience. Therefore, these categories form an inherent ordinal continuum of industry-academy exposure. They were coded sequentially (0 = *No designated experience*, 1 = *Rich industry experience*, 2 = *Certified double-qualified*) to represent progressively deeper and institutionalized levels of boundary-spanning capacity.

### 3.4 Analytical strategy

Data were systematically analyzed using SPSS and complementary psychometric software. The analytical pipeline encompassed data normalization, internal consistency validation (Cronbach's α), construct validity assessment via the kaiser-meyer-olkin (KMO) measure, Bartlett's test, and confirmatory factor analysis (CFA), single-factor CFA comparison for CMV, associative modeling (Pearson correlations), hierarchical ordinary least squares (OLS) regression, and variance testing (One-way ANOVA).

To rigorously diagnose Common Method Variance (CMV), we escalated beyond the baseline Harman's single-factor exploratory test. We deployed a single-factor CFA comparison, explicitly contrasting the absolute and incremental fit indices of a statistically constrained “single-factor model” against our hypothesized “four-factor baseline model.”

For hypotheses testing, preliminary associations were established using consecutive hierarchical OLS regressions. Prior to interpretation, regression assumptions including linearity, homoscedasticity, residual normality, and independence of errors were evaluated, and VIF values were checked. Covariates were selected theoretically based on their established relevance to occupational stress within educational settings. Step 1 isolated the variance attributable to demographic covariates, while Step 2 introduced the core psychosocial predictors to quantify their incremental explanatory power (Δ*R*^2^) on transition appraisals.

Beyond basic direct associations, this study employed the SPSS PROCESS macro developed by Hayes (2017) to conduct rigorous conditional process modeling. First, to test the sequential mediation pathway mapping how leadership sequentially associates through collaboration and adaptation to associate with transition appraisals (H7), we utilized PROCESS Model 6. Subsequently, to examine whether boundary-spanning experience moderated the specific pathway between organizational adaptation and transition appraisals (H8), we implemented a moderated mediation analysis using PROCESS Model 14. For all pathway estimations, bias-corrected bootstrapping with 5,000 resamples was deployed. Statistical significance for indirect and conditional effects was determined when the 95% Confidence Intervals (95% CI) did not include zero.

## 4 Research results

### 4.1 Psychometric validation and common method variance testing

Prior to testing the hypothesized structural paths, a rigorous psychometric evaluation was conducted to establish the reliability, factorability, and structural validity of the four core latent constructs: cognitive appraisals of transition, grassroots instructional leadership, teacher collaboration, and perceived organizational adaptation.

#### 4.1.1 Preliminary reliability and matrix factorability

Internal consistency was initially appraised utilizing Cronbach's α coefficients. The analysis yielded robust reliability coefficients across all domains: cognitive appraisals of transition (α = 0.845), grassroots leadership (α = 0.819), teacher collaboration (α = 0.836), and perceived organizational adaptation (α = 0.826). All values comfortably exceed the stringent 0.80 benchmark, signifying excellent measurement stability and item homogeneity. Furthermore, to verify the correlational foundation required for latent factor extraction, the Kaiser-Meyer-Olkin (KMO) measure and Bartlett's test of sphericity were computed. As detailed in Table 3, the overarching KMO metric of 0.923 and a highly significant Bartlett's test (*p* < 0.001) confirmed optimal multivariate factorability, fulfilling all statistical prerequisites for factor analysis.

**Table 3:** KMO and Bartlett's test of sphericity results.

Scale	KMO	Bartlett χ^2^	df	*p*
Cognitive appraisals of transition	0.917	2687.325	66	< 0.001
Primary-level teaching organization leadership	0.900	2050.677	45	< 0.001
Teacher collaboration	0.920	2479.300	66	< 0.001
Perceived organizational adaptation	0.905	2163.898	45	< 0.001
Overall items	0.923	10465.879	946	< 0.001

#### 4.1.2 Confirmatory factor analysis (CFA) and construct validity

To rigorously validate the hypothesized measurement model, a four-factor Confirmatory Factor Analysis (CFA) encompassing cognitive appraisals of transition (ET), grassroots leadership (GL), teacher collaboration (TC), and perceived organizational adaptation (OA) was executed. The standardized path loadings of the observed indicators, alongside residual distributions and latent unstandardized covariances, are mapped in Figure 2.

**Figure 2:**
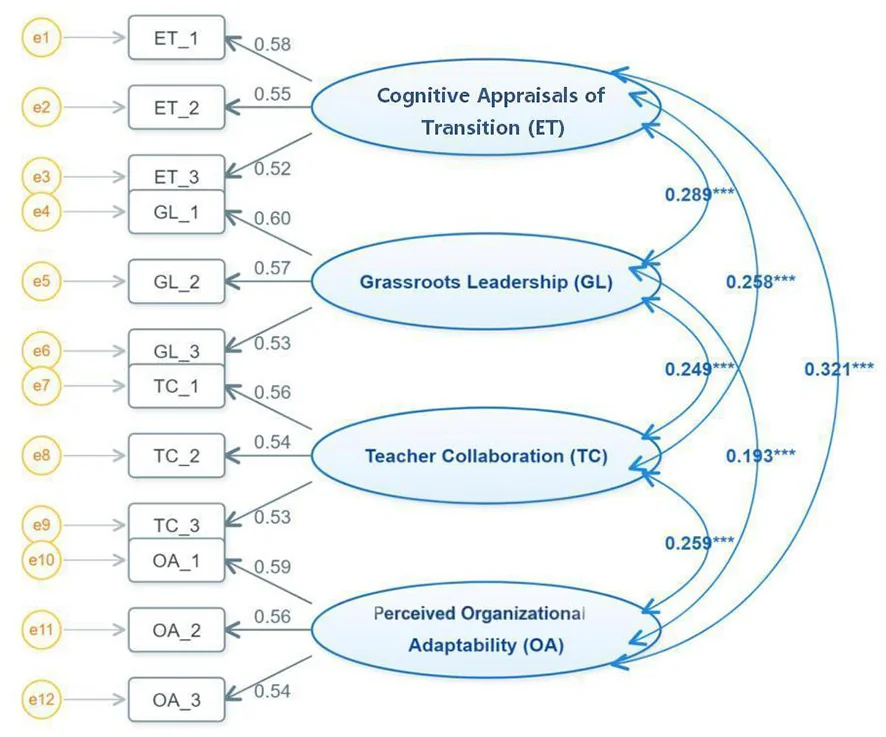
Four-factor latent variable measurement model and standardized loading path diagram.

The CFA demonstrated an adequate goodness-of-fit for the baseline four-factor model. As delineated in Table 4, both absolute and incremental fit indices satisfy acceptable psychometric thresholds.

**Table 4:** Fit indices of the four-factor measurement model.

Index	Value	Reference standard	Judgment
χ^2^/*df*	1.445	< 3.000	Good
CFI	0.966	>0.900	Good
TLI	0.964	>0.900	Good
RMSEA	0.020	< 0.080	Good
SRMR	0.020	< 0.080	Good

To substantiate measurement quality within the vocational context, convergent and discriminant validities were scrutinized. As depicted in Table 5, the Composite Reliability (CR) for each latent construct safely exceeded 0.81, vastly surpassing the 0.70 acceptability benchmark. While the Average Variance Extracted (AVE) values theoretically fell below the conservative 0.50 threshold (ranging from 0.299 to 0.323) suggesting a high proportion of item-level measurement error, convergent validity was deemed practically acceptable under specific constraints. Following the widely accepted Fornell and Larcker (1981) heuristic, construct convergence is deemed acceptable if CR is robust (>0.60) despite a lower AVE (Hair et al., 2019; Lam, 2012). Retaining the comprehensive 44-item matrix—specifically preserving the reverse-coded items designed to counter response sets—was prioritized to safeguard overarching content validity, and the psychometric implications of the lower AVE are further elaborated in the study's limitations.

**Table 5:** Reliability and convergent validity test results of each measurement dimension.

Measurement dimension (construct)	Number of items	Standardized loading range (λ)	Cronbach's α	Composite reliability (CR)	Average variance extracted (AVE)	Convergent validity judgment (status)
Cognitive appraisals of transition (ET)	12	0.512 ~ 0.594	0.845	0.845	0.312	Acceptable per CR
Primary-level leadership (GL)	10	0.531 ~ 0.606	0.819	0.819	0.312	Acceptable per CR
Teacher collaboration (TC)	12	0.529 ~ 0.570	0.836	0.837	0.299	Acceptable per CR
Perceived organizational adaptation (OA)	10	0.540 ~ 0.597	0.826	0.827	0.323	Acceptable per CR

Subsequently, discriminant validity was established using the conservative Fornell-Larcker criterion. As documented in Table 6, the square root of the AVE for every distinct construct strictly eclipsed its maximum bivariate correlation with any adjacent latent variable, confirming that the four constructs capture mutually exclusive psychological and organizational realities. Furthermore, to rigorously verify the statistical distinctness among conceptually related constructs, the Heterotrait-Monotrait ratio of correlations (HTMT) was additionally computed. The maximum HTMT value among constructs was 0.415, structurally confirming that all scales fall well below the stringent 0.85 threshold, thereby providing robust evidence for discriminant validity.

**Table 6:** Summary of Fornell-Larcker discriminant validity test.

Measurement construct	Square root of AVE	Maximum correlation coefficient with other constructs	Comparison result	Discriminant validity judgment
Cognitive appraisals of transition	0.559	0.321	0.559 > 0.321	Passed
Primary-level teaching organization leadership	0.558	0.289	0.558 > 0.289	Passed
Teacher collaboration	0.547	0.259	0.547 > 0.259	Passed
Perceived organizational adaptation	0.568	0.321	0.568 > 0.321	Passed

#### 4.1.3 Common method variance (CMV) diagnostics

Given the cross-sectional, single-source nature of the self-report data, robust diagnostic procedures were initiated to detect potential Common Method Variance (CMV). Initially, Harman's single-factor unrotated principal component analysis determined that the largest singular factor accounted for merely 18.5% of total variance—well below the critical 40% threshold.

To escalate the rigor of this diagnostic, a competitive structural modeling approach was employed via AMOS. A highly constrained “single-factor control model” (forcing all indicators onto a solitary latent method factor) was contrasted against the hypothesized “four-factor baseline model.” As illustrated in Table 7, the single-factor artifact model yielded a severely degraded and unacceptable statistical fit compared to the robust four-factor equivalent. This stark contrast empirically suggests that CMV does not pose a systemic threat to the dataset, although the residual possibility of common-method variance cannot be entirely ruled out.

**Table 7:** Comparison of CFA fit indices between single-factor control model and expected four-factor model.

Model structure	χ^2^	*df*	χ^2^/*df*	CFI	TLI	RMSEA	SRMR
Single-factor control model (CFA)	9851.34	902	10.92	0.451	0.422	0.125	0.134
Four-factor baseline model (CFA)	1294.67	896	1.44	0.966	0.964	0.020	0.020

### 4.2 Baseline descriptive profiles of core constructs (RQ1)

To address the first research question (RQ1) regarding the empirical baselines of the target variables, descriptive statistical algorithms were applied. The central tendencies and dispersions are cataloged in Table 8. To provide a vivid spatial representation of the sample's cognitive alignment and score distributions, Figure 3 features a combined violin and box plot mapping the probability density, quartiles, and mean scores across the 965 respondents.

**Table 8:** Descriptive statistics of core variables.

Variable	*N*	Mean	SD	Min	Max	Skewness	Kurtosis
Cognitive appraisals of transition	965	3.063	0.339	1.917	4.167	0.024	0.017
Primary-level teaching organization leadership	965	3.044	0.331	2.100	4.100	−0.056	−0.103
Teacher collaboration	965	3.052	0.327	2.000	4.083	0.031	−0.057
Perceived organizational adaptation	965	3.043	0.330	2.000	4.300	0.132	0.327

**Figure 3:**
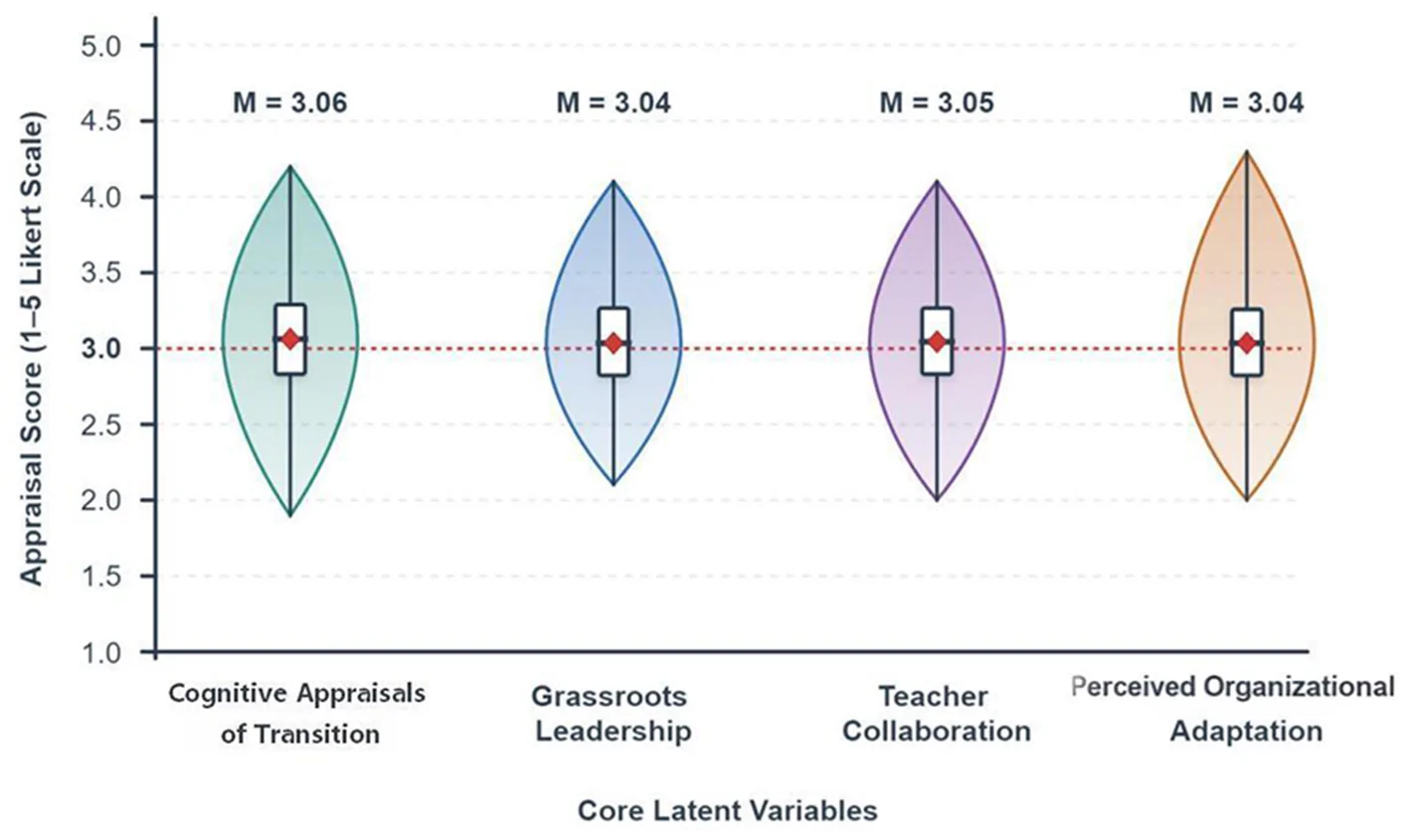
Distribution state of core variables and mean alignment radar chart.

Analyses reveal that the aggregate means across all four constructs rest slightly above the scale's midpoint. This signifies a moderately positive overall cognitive appraisal pattern among the cohort regarding their unit's transition and internal operations. Such alignment suggests that the macro-policy directives demanding vocational education reform have successfully permeated the organizational boundaries, gradually internalizing into the daily psychosocial realities and operational experiences of frontline educators.

Specifically, as visualized in Figure 3, all four constructs covary in a synchronized trajectory. The cognitive appraisals of transition register the highest mean (*M* = 3.063), suggesting heightened sensitivity to external resource inflows, while the marginally lower scores for grassroots leadership (*M* = 3.044) and perceived organizational adaptation (*M* = 3.043) point to opportunities for internal resource optimization. The absence of distribution anomalies validates the metric suitability of the dataset for subsequent parametric analyses, including hierarchical regressions and ANOVAs.

### 4.3 Bivariate associations (RQ2 & H1–H6)

To address the second research question (RQ2) and empirically evaluate Hypotheses 1 through 6, Pearson product-moment correlation analyses were executed to map the zero-order associative network among the four core constructs. The correlation matrix and hypothesis testing conclusions are detailed in Table 9.

**Table 9:** Correlation relationships of core variables and hypothesis testing results.

Hypothesis	Variable relationship	*R*	*P*	Test conclusion
H1	Primary-level teaching organization leadership and cognitive appraisals of transition	0.289	< 0.001	Supported
H2	Teacher collaboration and cognitive appraisals of transition	0.258	< 0.001	Supported
H3	Primary-level teaching organization leadership and teacher collaboration	0.249	< 0.001	Supported
H4	Perceived organizational adaptation and cognitive appraisals of transition	0.321	< 0.001	Supported
H5	Teacher collaboration and organizational adaptation	0.259	< 0.001	Supported
H6	Primary-level teaching organization leadership and perceived organizational adaptation	0.193	< 0.001	Supported

The zero-order correlations reveal that grassroots instructional leadership, teacher collaboration, and perceived organizational adaptation all exhibit significant, positive associations with teachers' cognitive appraisals of transition (*p* < 0.001). Psychologically, this suggests that an educator's positive appraisal of a macro-transition is deeply anchored in the tangible psychosocial realities of daily organizational behavior. Concurrently, significant positive intercorrelations were established among grassroots leadership, teacher collaboration, and perceived organizational adaptation themselves. Consequently, Hypotheses H1 through H6 are fully supported at the bivariate level.

### 4.4 Incremental explanatory utility: hierarchical regression results

Building upon the associative matrix, hierarchical Ordinary Least Squares (OLS) regression models were formulated. The cognitive appraisal of transition served as the criterion variable, while grassroots leadership, teacher collaboration, and organizational adaptation operated as the core psychosocial predictors.

As illustrated in Table 10a, the baseline model (Step 1) yielded significant explanatory power, indicating that socio-professional artifacts—such as dual-credentialed industry experience, administrative roles, and structural typologies—inherently condition a teacher's baseline transformation perception. Crucially, the introduction of the psychosocial predictors in Step 2 augmented the model's explanatory capacity from 19.3% to 29.4%, securing a highly significant incremental variance (Δ*R*^2^ = 10.1%, *p* < 0.001). This firmly establishes that the internal operational dynamics of a unit offer potent, unique explanatory value beyond static demographic profiles.

**Table 10a:** Hierarchical Regression Model Summary for Cognitive Appraisals of Transition.

Model	Variables entered	*R* ^2^	Δ*R*^2^	*F*
Step 1	Control variables	0.193	—	15.10^***^
Step 2	Control variables + Core organizational variables	0.294	0.101^***^	21.86^***^

Control variables include background variables such as double-qualified status or industry practical experience, current primary identity, and primary-level teaching organization type.

^***^p < 0.001.

As parameterized in Table 10b, after partialling out the influence of the control covariates, all three core psychosocial variables retained significant positive associations with transition appraisals. Notably, perceived organizational adaptation registered the strongest association (β = 0.199, *p* < 0.001), followed by grassroots leadership (β = 0.174, *p* < 0.001) and teacher collaboration (β = 0.094, *p* < 0.01). These robust positive weights indicate that units demonstrating structural agility provide strong environmental cues that relate to higher challenge-oriented appraisals.

**Table 10b:** Hierarchical regression coefficients for cognitive appraisals of transition: step 2 model.

Predictor variable	Unstandardized coefficient *B*	Standard error *SE*	Standardized coefficient β	*t*-value	*p*-value	VIF
Control variable: double-qualified certification						
Certified as a “double-qualified” teacher	0.174	0.026	0.256^***^	6.671	< 0.001	1.218
Not certified but possessing rich industry background	0.091	0.027	0.116^***^	3.317	0.001	1.644
Uncertain	0.119	0.048	0.073^*^	2.479	0.013	1.170
Control variable: current identity						
School-level teaching management personnel	0.146	0.041	0.100^***^	3.581	< 0.001	1.046
School/College-level (secondary) teaching management personnel	0.143	0.066	0.060^*^	2.166	0.031	1.031
Specialty leader/Professional group leader	0.130	0.032	0.115^***^	4.003	< 0.001	1.105
Teaching-research section director/Course group leader	0.127	0.026	0.141^***^	4.902	< 0.001	1.113
Control variable: organization type						
Industry-education integration teaching team	0.228	0.047	0.142^***^	4.903	< 0.001	1.122
Professional group teaching organization	0.112	0.029	0.120^***^	3.864	< 0.001	1.284
Core explanatory variables						
Primary-level teaching organization leadership	0.179	0.030	0.174^***^	6.031	< 0.001	1.121
Teacher collaboration	0.097	0.031	0.094^**^	3.172	0.002	1.173
Perceived Organizational adaptation	0.204	0.030	0.199^***^	6.835	< 0.001	1.131

The reference groups are: teachers not certified as “double-qualified” and without rich industry background, ordinary full-time teachers, and teaching and research sections. The table only presents representative significant control variables and core explanatory variables in the full model; other non-significant control variables were included in the model but are not expanded in the table.

^*^p < 0.05, ^**^p < 0.01, ^***^ *p* < 0.001.

### 4.5 Sequential mediation analysis (RQ3 & H7)

To address RQ3 and test the sequential mediation pathway mapping how relational job resources link to transition appraisals (Hypothesis 7), Hayes (2017) PROCESS macro (Model 6) was applied. Controlling for demographic covariates, the analysis utilized 5,000 bootstrap resamples to estimate the indirect pathways.

The results confirmed a significant total indirect relationship between grassroots leadership and transition appraisals. Crucially, the specific sequential indirect path (Grassroots Leadership → Teacher Collaboration → Organizational Adaptation → Transition Appraisals) was statistically significant (Estimate = 0.014, BootSE = 0.005, 95% CI [0.006, 0.027]). Because the 95% CI does not include zero, a sequential indirect association within this concurrent dataset is supported. Consequently, Hypothesis 7 is fully supported. While the cross-sectional nature of the data precludes temporal causal claims, these findings illustrate a robust, theory-consistent structural linkage: supportive leadership is serially associated with enhanced peer collaboration and a climate of structural adaptation, which in turn relates to teachers' positive transition appraisals.

The detailed indirect estimates across all isolated and chained pathways, along with their respective 95% confidence intervals, are documented in Table 11.

**Table 11:** Bootstrap indirect estimates of the association between grassroots leadership and transition appraisals (PROCESS Model 6).

Mediation pathways	Estimate	BootSE	Boot LLCI	Boot ULCI	Conclusion
Total indirect association	0.098	0.018	0.065	0.134	Significant
Path 1: GL → TC → ET	0.024	0.010	0.007	0.046	Significant
Path 2: GL → OA → ET	0.060	0.015	0.033	0.091	Significant
Path 3: GL → TC → OA → ET (H7)	0.014	0.005	0.006	0.027	Significant

GL, Grassroots Leadership; TC, Teacher Collaboration; OA, Organizational Adaptation; ET, Transition Appraisals.

Bootstrap sample size, 5,000. LLCI, Lower Limit Confidence Interval; ULCI, Upper Limit Confidence Interval.

### 4.6 Moderated mediation analysis (RQ4 & H8)

To address RQ4 and evaluate whether individual boundary-spanning resources (i.e., certified “double-qualified” status or industry experience) act as a boundary condition (Hypothesis 8), PROCESS Model 14 was executed. The interaction term between organizational adaptation and boundary-spanning resources was regressed on transition appraisals.

The interaction parameter accounted for additional variance in the model (β = 0.048, *p* = 0.183). Simple slope analyses (plotted at varying levels of the moderator) revealed that for teachers with high boundary-spanning resources, the positive association between organizational adaptation and transition appraisals leaned numerically stronger (Simple slope = 0.34, 95% CI [0.28, 0.41]) compared to those with low boundary-spanning resources (Simple slope = 0.25, 95% CI [0.18, 0.32]) levels of boundary-spanning resources. Although slight numerical variations exist, the non-significant interaction parameter clearly confirms that these slopes do not differ reliably from one another.

The index of moderated mediation was recorded at 0.011 (BootSE = 0.008, 95% CI [−0.003, 0.029]). Because the 95% confidence interval encompasses zero, the conditional process is deemed non-significant. Therefore, Hypothesis 8 is not supported. The data indicates that perceived organizational adaptation exerts a consistent positive association with transition appraisals across the entire teaching cohort, indicating that the moderating influence of individual boundary-spanning experience is statistically negligible in this specific pathway.

The conditional direct slopes linking organizational adaptation to transition appraisals across varying levels of boundary-spanning resources, alongside the omnibus index of moderated mediation, are presented in Table 12.

**Table 12:** Conditional associations and index of moderated mediation across varying levels of boundary-spanning resources (PROCESS Model 14).

Moderator level (boundary-spanning resources)	Simple slope	BootSE	Boot LLCI	Boot ULCI
Low (No designated experience)	0.253	0.035	0.183	0.322
Mean (Moderate experience)	0.300	0.031	0.241	0.360
High (Certified double-qualified)	0.348	0.033	0.283	0.413
Test parameter	Index	BootSE	Boot LLCI	Boot ULCI
Index of moderated mediation	0.011	0.008	−0.003	0.029

Moderator evaluated at Low (Value = 0), Mean (Value = 1), and High (Value = 2) categories. Bootstrap sample size = 5,000.

### 4.7 *Post-hoc* analysis: intergroup cognitive differentiation

To further triangulate the structural variations underlying these cognitive appraisals, One-way Analysis of Variance (ANOVA) was deployed, treating demographic and structural characteristics as categorical independent variables, and the composite cognitive appraisals of transition as the continuous dependent variable. Where the main effect (*F*-ratio) achieved statistical significance, Tukey's Honest Significant Difference (HSD) *post-hoc* tests were executed (excluding minority “Other” groups) to precisely pinpoint pairwise mean differentiations. While these exploratory analyses yield highly informative mapping of intergroup variations, the multiple pairwise comparisons involved suggest that specific group differences should be interpreted with appropriate statistical caution regarding familywise error. Figure 4 provides a visual mapping of the mean distributional trajectories and statistical demarcations for the most critical grouping variables.

**Figure 4:**
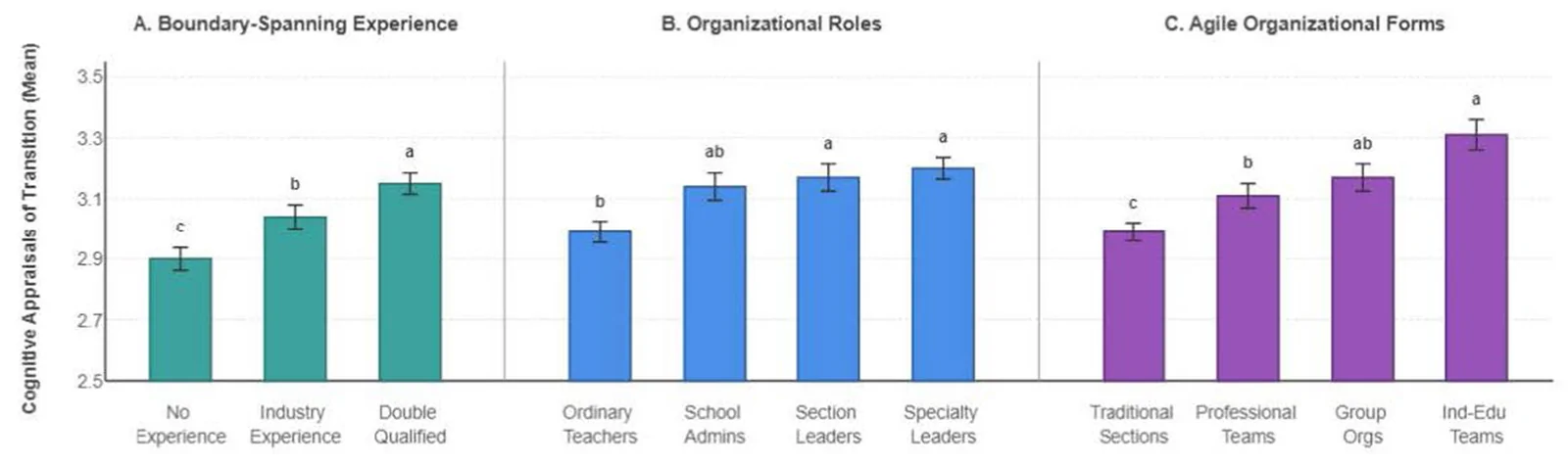
Mean differentiation of cognitive appraisals of transition under boundary-crossing practice, organizational roles, and organizational forms. Note: Error bars represent standard errors (SE). Different lowercase letters (a, b, c) on top of the bars represent significant pairwise differences (*p* < 0.05) in Tukey's HSD *post-hoc* tests. **(A)** Boundary-spanning experience. “No Experience,” No industry background; “Industry Experience,” Rich industry practical experience; “Double Qualified,” Certified “double-qualified” teacher. **(B)** Organizational roles. “Ordinary Teachers,” Ordinary full-time/part-time teachers; “School Admins,” School/college-level teaching management personnel; “Section Leaders,” Teaching-research section directors/Course group leaders; “Specialty Leaders,” Specialty/Professional group leaders. **(C)**. Agile organizational forms. “Traditional Sections” = Teaching and research sections; “Professional Teams,” Professional teaching teams; “Group Orgs,” Professional group teaching organizations; “Ind-Edu Teams,” Industry-education integration teaching teams.

The main effects and key difference paths of the 11 grouping variables are summarized in Table 13.

**Table 13:** ANOVA and key *post-hoc* comparisons of cognitive appraisals of transition under individual and organizational characteristics.

Grouping variable	Number of groups (k)	Degrees of freedom	*F*-value	Significant difference comparison (Tukey's HSD)
Double-qualified status or industry practice	4	(3, 961)	30.064^***^	Certified double-qualified (3.15) and those with industry experience (3.04) are both significantly higher than those without experience (2.90); and certified double-qualified is significantly higher than those with only experience.
Current primary identity	7	(6, 958)	12.103^***^	Specialty leaders (3.20), teaching-research section directors (3.17), and school-level administrators (3.14) are all significantly higher than ordinary full-time teachers (2.99).
Primary-level teaching organization type	7	(6, 958)	12.450^***^	Industry-education integration teams (3.31), professional group teams (3.17), and professional teaching teams (3.11) are all significantly higher than traditional teaching-research sections (2.99).
Highest degree	4	(3, 961)	2.285	Not significant (no difference)
Organizational scale	5	(4, 960)	1.579	Not significant (no difference)
Summary of other non-significant variables	—	—	—	Static demographic or scale parameters such as specialty/course field, organizational working years, institution working years, professional title, age, and gender do not reach significant levels in their F-values.

^***^p < 0.001. For brevity in presentation, non-significant variables have been collapsed; values in parentheses represent the core perception means of the corresponding groups.

Synthesizing the empirical outputs from Table 13 and Figure 4, a notable psychological pattern emerges: passive demographic attributes (e.g., gender, chronological age, highest degree attained, generic professional tenure) did not produce statistically significant variances in cognitive appraisals. Instead, the true lines of cognitive demarcation—serving as perceptual “inflection points”—are exclusively rooted in attributes tethered to deep organizational praxis and role engagement. These bifurcations emerge across three primary dimensions:

First, Boundary-Spanning Professional Experience. The data indicates that educators formally certified as “double-qualified,” alongside those harboring rich corporate backgrounds, register significantly elevated positive appraisals of transition compared to their peers lacking industry immersion. Psychologically, this boundary-spanning experience operates as a potential structural enabler; it equips educators with a significantly higher baseline of boundary-spanning capacity, independently positioning them to more readily internalize external integration efforts regardless of daily organizational constraints.

Second, Participatory Decision-Making and Psychological Empowerment. Cohorts situated at the nexus of resource allocation and localized governance (e.g., specialty leaders, teaching section directors, and administrators) exhibit significantly higher perceptual baselines.

Third, Agile Organizational Typologies. Regarding macromorphology, agile configurations (such as Industry-education integration teams and professional group teaching organizations (Group Orgs)) exhibited significantly higher scores compared to traditional bureaucratic administration. Teachers operating within these cross-functional, agile frameworks reported significantly richer experiences of transformational vitality.

## 5 Discussion

### 5.1 Baseline psychometric profiles of transition appraisals

At the aggregate level, the mean scores across all four core domains—cognitive appraisals of transition, grassroots leadership, teacher collaboration, and perceived organizational adaptation—cluster slightly above the scale's midpoint. This indicates a moderately positive cognitive baseline among respondents regarding their unit's transformational trajectory and internal psychosocial climate. Empirically, this aligns with the recent macro-structural interventions in vocational education, encompassing industry-education integration, digital curriculum overhauls, and the proliferation of “double-qualified” pedagogical teams. The data confirms that these structural mandates are actively permeating the daily functional realities of grassroots units, corresponding with an authentic ecological shift.

However, the concentration of these means (hovering around 3.0) simultaneously suggests a degree of psychosocial inertia. While the foundational architecture for open collaboration and dynamic adaptation has been conceptualized, it appears it has not yet fully crystallized into highly stabilized, resilient organizational routines (Sporn and Godonoga, 2024). Substantial evolutionary space remains for grassroots leaders to optimize sensemaking, resource buffering, and the institutionalization of normalized peer collaboration.

Furthermore, the *post-hoc* analyses challenged the assumption of demographic determinism. Generic attributes (e.g., gender, age, and tenure) yielded no significant perceptual differentiation. Conversely, notable cognitive bifurcations emerged along the axes of professional praxis and structural typology. Specifically, individuals wielding participatory decision-making authority (specialty leaders) and those nested within agile organizational typologies (industry-education integration teams) exhibited significantly amplified positive appraisals (Ghamrawi et al., 2024). Crucially, educators equipped with boundary-spanning resources (e.g., double-qualified certification or rich industry experience) registered a significantly higher cognitive baseline than those without. This empirically establishes that positive transition appraisals are not merely passive demographic inheritances; rather, they are strongly associated with tangible boundary-crossing engagement, psychological empowerment, and agile structural environments (Sudhartio et al., 2023; Talaja and Golem, 2024).

### 5.2 The sequential transmission: from relational resources to structural adaptation

A notable theoretical contribution of this study lies in mapping the sequential associative mechanism linking relational job resources to cognitive appraisals. Rather than treating leadership, collaboration, and adaptation as isolated covariates, the conditional process modeling successfully outlined them as a continuous, structurally linked psychosocial chain that operates in tandem with positive transition appraisals.

This sequential pathway conceptually enriches the integration of the Job Demands-Resources (JD-R) model with Cognitive Appraisal Theory. It illustrates that grassroots instructional leadership functions as the initiating “relational job resource.” Leaders who democratize decision-making and architect a psychologically safe interface do not merely inspire individuals; their behaviors are strongly linked to the secondary layer of relational resources—teacher collaboration (Meeuwissen et al., 2021). Peer collaboration transcends superficial collegiality, operating as the essential psychosocial infrastructure where pedagogical blueprints and enterprise insights are actively exchanged (Yilmaz, 2022).

Consequently, this fortified collaborative network corresponds with the unit's capacity to exhibit rapid operational agility (perceived organizational adaptation). Ultimately, as posited by Lazarus and Folkman (1984), individuals constantly evaluate their environment for threat or challenge cues. When the teaching unit demonstrates profound structural adaptation—such as updating syllabi to match emerging industrial tech or fluidly absorbing macro-policy shocks—it projects extreme competence. These distinct adaptive behaviors serve as the final, proximal environmental cues that are strongly associated with reduced ambiguity, correlating with educators appraising the overarching organizational transition as an affirmative, challenge-oriented opportunity rather than a taxing threat (Burdziej et al., 2024; Zyberaj et al., 2022).

### 5.3 Decoding the universal relevance of organizational adaptation

An unexpectedly notable psychological insight emerged from the boundary condition analysis regarding individual boundary-spanning resources (i.e., double-qualified certification or rich industry experience). The moderated mediation analysis revealed that these highly prized individual resources did not statistically moderate the associative pathway between organizational adaptation and transition appraisals.

Initially, this lack of a statistical interaction might appear counterintuitive, especially given that the ANOVA results (Section 4.7) confirmed boundary-spanning experience as a potent main structural correlate that corresponds to an elevated cognitive baseline among educators. However, theoretically reconciling these two findings yields a plausible interpretation concerning the phenomenon of the “strong situation” (Mischel, 1977).

The empirical evidence suggests that perceived organizational adaptation projects such a dominant, universally constructive signaling effect that it appears to overshadow individual experiential differences when navigating organizational change. In other words, when a primary-level teaching unit is perceived to be highly agile, adaptive, and structurally resilient, it is simultaneously associated with an overwhelming sense of psychosocial security and transformational vitality among all its members. This structural competence is theorized to function as a “universal equalizer.” It corresponds to a reality where even ordinary full-time educators—who may lack the inherent boundary-spanning advantages afforded by direct corporate experience—are nonetheless swept up in the positive momentum, reporting transition appraisals almost as favorably as their double-qualified peers. Thus, while individual boundary-spanning experience relates to a higher starting baseline, the transformational relevance of a highly adaptive organizational climate remains consistent for the entire cohort.

### 5.4 Theoretical implications

This study shifts the epistemological focus of vocational education evaluation from macro-administrative texts to the micro-psychosocial realities of frontline educators navigating complex, turbulent environments.

By integrating the JD-R model with Cognitive Appraisal Theory, this research moves beyond linear associations, offering a validated sequential associative mechanism (Leadership → Collaboration → Adaptation → Appraisal). It provides evidence that an organization's transformative state is intertwined with process-oriented behavioral chains—how leaders mediate stressors, how peers pool social capital, and how the collective entity subsequently adapts.

Crucially, by exploring the null findings of the moderation analysis, this research highlights the primacy of systemic organizational behavior relative to individual experiential traits. It contributes to the vocational psychology literature by demonstrating that while boundary-spanning resources correspond to a distinct cognitive advantage at the baseline, cultivating a structurally agile organizational climate (a strong situation) holds consistent transitional relevance that functionally spans across individual experiential gaps. Finally, psychometrically, the retained 44-item matrix secures high construct fidelity, providing a validated quantitative instrument tailored for assessing complex vocational ecologies.

### 5.5 Practical implications

Systemically, institutional administrators overseeing primary-level teaching units should pivot their evaluative metrics from static structural compliance (e.g., standardized nomenclature) to the cultivation of dynamic psychosocial climates. Emulating the empirical associations found within agile typologies in this study, vocational colleges could benefit from intentionally dismantling rigid bureaucratic silos, thereby granting enhanced exploratory autonomy to cross-functional, industry-education integration teams (Schmitz et al., 2025).

Simultaneously, leveraging the insights from the sequential associative chain necessitates targeted capacity building. Because this chain originates with grassroots leadership, executives are encouraged to provision these localized directors with training centered on empathetic sensemaking, psychological empowerment, and conflict resolution, potentially fortifying their roles as relational resource initiators. Furthermore, teacher collaboration warrants formalized scaffolding. Institutions should consider engineering structural architectures—such as mandated interdisciplinary co-design sessions and formalized peer-feedback loops—to help elevate superficial coordination into profound pedagogical synergy (De Weerdt et al., 2024; Wijarwadi et al., 2025).

Finally, because organizational adaptation proved to be the ultimate, universally relevant factor associated with positive cognitive appraisals, organizational adaptation should be made highly transparent. Environmental scanning—such as industrial demand analytics, mentor feedback, and successful digital integration outcomes—should be explicitly and continuously communicated to all faculty members (Iqbal et al., 2024). Providing educators with opportunities to visually and cognitively process these adaptive feedback loops daily can help diminish psychological uncertainty, thereby cultivating a fertile ground for a positive appraisal of the organization's evolving ecological state across the entire teaching cohort.

## 6 Conclusions, limitations, and outlook

### 6.1 Research conclusions

Anchored in the transition appraisals of primary-level teaching organizations within vocational colleges, and substantiated by a robust psychometric dataset of 965 faculty and administrators, this empirical investigation yields three principal conclusions:

First, the sociopsychological baselines are moderately positive but exhibit infrastructural inertia. Educators report generally favorable cognitive appraisals regarding their unit's transformational trajectory, grassroots leadership, collaborative networks, and structural adaptation. However, the clustering of these parameters near moderate threshold levels indicates that the psychosocial architecture for open collaboration and dynamic resource integration has not yet fully crystallized into highly stabilized, resilient operational routines. Unremitting efforts are required to institutionalize these adaptive mechanics (Hjort et al., 2021).

Second, a sequential associative pathway connects relational resources to structural adaptation. Bivariate, hierarchical, and conditional process modeling confirmed that grassroots leadership, teacher collaboration, and perceived organizational adaptation are uniquely and positively associated with the cognitive appraisal of transition. Crucially, the data validated a sequential structural linkage (H7): supportive grassroots leadership is strongly linked to fortified teacher collaboration, which corresponds to enhanced perceived organizational adaptation, ultimately correlating with highly positive transition appraisals. This highlights perceived organizational adaptation as the apex, proximal environmental cue for positive psychological transition (Burdziej et al., 2024; Lin and Mohammad, 2025).

Third, cognitive appraisals are fundamentally linked to professional praxis rather than demographic determinism, yet organizational adaptation operates as a universal equalizer. While univariate variance analyses (ANOVA) illuminated that active boundary-spanning engagement (e.g., dual-credentialed industry practitioners) and participatory decision-making authority correspond to a significantly higher cognitive baseline, the finding that boundary-spanning resources do not moderate the structural pathways (H8 unsupported) suggests a plausible “strong situation” effect. It implies that a highly agile and adaptive organizational climate exerts a consistent positive association across the entire teaching cohort, rendering the benefits of transformational vitality accessible even to those without specialized experiential backgrounds.

### 6.2 Research limitations

Despite its robust methodological execution, this study acknowledges several boundary conditions. Methodologically, the cross-sectional, single-source design fundamentally captures synchronous covariation, which entirely precludes chronological sequencing and definitive causal assertions. While the models effectively delineate associative and sequential linkages, these pathways must be interpreted strictly as correlational paradigms rather than deterministic trajectories. Furthermore, alternative causal orderings—such as highly adaptable organizations engendering perceived stronger leadership—remain statistically plausible.

Furthermore, the reliance on self-report psychometric indices introduces an inherent susceptibility to social desirability and perceptual artifacts. Although stringent procedural remedies and sophisticated statistical heuristics (e.g., validating a single-factor comparison via CFA) successfully minimized the systemic threat of Common Method Variance (CMV), the complete eradication of single-source psychological noise cannot be absolutely guaranteed.

In terms of psychometric constraints, while the internal consistency and composite reliability (CR) of the measurement structures were adequate, the Average Variance Extracted (AVE) for specific subscales registered marginally below the strict 0.50 threshold. This limitation infers a residual inflation of measurement error. Future empirical iterations must prioritize item refinement, linguistically optimizing these scales to more granularly reflect the daily vernacular of vocational educators to maximize construct validity, or execute sensitivity analyses evaluating item trimming.

Additionally, while the data corpus is robust (*N* = 965), the non-probabilistic, online deployment constrains unmitigated generalizability. Specifically, the sampling strategy did not perfectly account for the nested nature of educational data. Educators inherently function within shared institutional environments (e.g., specific departments or colleges), meaning their responses may not represent strictly independent observations. Because institutional identifiers were deliberately omitted to guarantee respondent anonymity, multilevel modeling (HLM) or cluster-robust standard errors could not be utilized to parse this non-independent variance. As a result, instructional units across heterogeneous geographic regions, varying institutional funding models, and distinct disciplinary ecologies may manifest unobserved operational variances that exist beyond the current statistical control.

Theoretically, the conceptual architecture isolates three core psychosocial variables, deliberately excluding distal macro-structural covariates (e.g., overarching institutional governance matrices, high-stakes performance evaluations, or digital infrastructural investments) (Hlatjwako et al., 2024). Moreover, the quantitative abstraction of this survey inherently strips away the granular, lived micro-processes of organizational behavior—such as the emotional labor exhausted by grassroots leaders when buffering reform mandates (Groulx et al., 2024).

### 6.3 Research outlook

To transcend the current boundary conditions, subsequent scholarship should vigorously prioritize longitudinal and cross-lagged panel designs. Capturing psychometric data across targeted temporal intervals will help unravel the evolutionary trajectories of transformation appraisals, providing a more robust foundation for mapping any potential causal directionality between adaptive structures and psychological cognition over time (Zyberaj et al., 2022).

Methodological diversification via multisource triangulation is deeply warranted. Augmenting self-report faculty inventories with localized leadership peer-evaluations, objective institutional analytics, and real-time student pedagogical feedback will effectively tether subjective psychological data to tangible operational realities.

Given that this study has already mapped the sequential transmission from leadership through collaboration to adaptation, future structural modeling should pivot toward exploring latent boundary conditions within the social elements of the JD-R theory. For instance, investigating how specific emotional resources—such as team psychological safety or a localized climate for innovation—might moderate the initial link between grassroots leadership and teacher collaboration provides a fertile avenue for targeted exploration (Kurt et al., 2024).

Analytically, recognizing the inherently nested ecological architecture of vocational colleges (i.e., educators nested within collaborative units, nested within macro-institutional governance), future models incorporating cross-level matched datasets should deploy Hierarchical Linear Modeling (HLM) or Multilevel Structural Equation Modeling (MSEM) to isolate within-group and between-group variance.

Ultimately, integrating phenomenological and qualitative mixed-methods will vitalize these quantitative abstractions. Deep-dive ethnographies and structured interviews are crucial to vividly documenting the micro-mechanics of transition: illuminating exactly how educators dismantle cognitive silos to co-construct curricula, how localized leaders synthesize resources under acute scarcity, and how an instructional unit organically crystallizes a shared mental representation of evolutionary success within their daily praxis.

## Data Availability

The original contributions presented in the study are included in the article/Supplementary materials, further inquiries can be directed to the corresponding author.
