# Supplementary material for: Shaping teachers' challenge appraisals of organizational transition: the roles of grassroots leadership, peer collaboration, and boundary-spanning experience
Source: Front Psychol. 2026 Oct 7;17:1949606. doi: 10.3389/fpsyg.2026.1949606 (PMC13682965; doi:10.3389/fpsyg.2026.1949606)
Supplement: Supplementary file 1 [file Data_Sheet_1.docx]

Supplementary Material S1: Questionnaire on the Ecological Transformation of Primary-level Teaching Organizations in Vocational Colleges

**S1.1 Informed Consent**

Dear Teachers,

Hello! This questionnaire is only intended for individuals who are at least 18 years old and are currently engaged in work related to teaching, teaching and research, curriculum construction, specialty construction, or teaching management in vocational colleges. The questionnaire is anonymous and does not collect personally identifiable information such as name, ID number, mobile phone number, employee ID, or specific institution name. Your participation is entirely voluntary, and you may unconditionally withdraw from filling it out at any time. All data will be used solely for academic research and will be presented as aggregated statistical results.

If you have read and understood the above content and agree to participate in this study, please select:

[ ] I have read and agree to participate in this study

[ ] I do not agree to participate in this study (If you choose this option, please stop filling out the questionnaire)

**S1.2 Eligibility Check**

Please confirm whether you meet the following conditions:

You are at least 18 years old;

You are currently engaged in teaching, teaching and research, curriculum construction, specialty construction, or teaching management related work in a vocational college;

You voluntarily participate in this study.

Please select:

[ ] Yes, I meet the above conditions and agree to continue filling out the questionnaire

[ ] No, I do not meet the above conditions or do not agree to continue filling out the questionnaire (If you choose this option, please stop filling out the questionnaire)

**S1.3 Instructions**

The “primary-level teaching organization” in this study refers to the primary-level organizational unit in your vocational college that directly undertakes tasks such as teaching, teaching and research, curriculum construction, specialty construction, talent cultivation, and teaching reform (such as teaching and research sections, course groups, professional teaching teams, professional group teaching teams, teaching innovation teams, etc.). When answering, please evaluate based on the primary-level teaching organization that you are most familiar with and have the highest level of participation in.

The evaluation uses a 5-point Likert scale: 1 = Strongly disagree, 2 = Disagree, 3 = Neutral/Uncertain, 4 = Agree, 5 = Strongly agree.

**S1.4 Part I: Demographic Background**

Your gender:

[ ] Male [ ] Female [ ] Other/Prefer not to say

Your age:

[ ] 18–30 years old [ ] 31–40 years old [ ] 41–50 years old [ ] 51 years old and above [ ] Prefer not to answer

Your highest degree:

[ ] Junior college and below [ ] Bachelor’s degree [ ] Master’s degree [ ] Doctoral degree

Your professional title:

[ ] Unrated [ ] Primary (Junior) [ ] Intermediate [ ] Associate senior [ ] Senior

Your years of working in vocational colleges:

[ ] 3 years and below [ ] 4–10 years [ ] 11–20 years [ ] 21 years and above

Your years of working in the current primary-level teaching organization:

[ ] Less than 1 year [ ] 1–3 years [ ] 4–6 years [ ] 7–10 years [ ] More than 10 years

Your current primary identity in the school:

[ ] Full-time teacher (no administrative or team position) [ ] Part-time teacher [ ] Teaching-research section director/Course group leader [ ] Specialty leader/Professional group leader [ ] School/College-level (secondary) teaching management personnel [ ] School-level teaching management personnel [ ] Other

The type of primary-level teaching organization you are evaluating (Single choice):

[ ] Teaching and research section [ ] Course group [ ] Professional teaching team [ ] Professional group teaching organization [ ] Teacher teaching innovation team [ ] Industry-education integration teaching team [ ] Other

The approximate scale of the primary-level teaching organization you are evaluating:

[ ] 5 people and below [ ] 6–10 people [ ] 11–20 people [ ] 21–30 people [ ] 31 people and above

The main broad field corresponding to your specialty or course:

[ ] Equipment Manufacturing [ ] Electronic Information [ ] Finance and Trade [ ] Transportation [ ] Medicine and Health [ ] Education and Sports [ ] Culture and Art [ ] Tourism [ ] Agriculture, Forestry, Animal Husbandry, and Fishery [ ] Civil Engineering and Architecture [ ] Public Administration and Service [ ] Other

Do you have a “double-qualified” teacher status or related industry practice experience?:

[ ] Yes, officially certified as a “double-qualified” teacher

[ ] Yes, not certified, but have rich industry practice experience (e.g., cumulative enterprise practice of more than 6 months)

[ ] No

[ ] Uncertain

**S1.5 Part II: Measurement Scales**

1. Cognitive Appraisals of Transition Scale (ET)

| Code | Items | Strongly disagree (1) | Disagree (2) | Neutral/Uncertain (3) | Agree (4) | Strongly agree (5) |
| --- | --- | --- | --- | --- | --- | --- |
| ET1 | Our organization can promptly pay attention to changes in vocational education policies and talent cultivation needs. | [ ] | [ ] | [ ] | [ ] | [ ] |
| ET2 | The developmental positioning of our organization can closely align with the actual needs of regional industries. | [ ] | [ ] | [ ] | [ ] | [ ] |
| ET3 | Our organization has formed a clear characteristic positioning in specialty construction and curriculum system restructuring. | [ ] | [ ] | [ ] | [ ] | [ ] |
| ET4 | Our organization can maintain stable, multi-dimensional industry-university-research contacts with external enterprises and public institutions. | [ ] | [ ] | [ ] | [ ] | [ ] |
| ET5 | Our organization can effectively integrate on-campus and off-campus experimental and practical training bases and industry mentor resources. | [ ] | [ ] | [ ] | [ ] | [ ] |
| ET6 | The internal barriers between specialties or classes/groups in our organization are strong, and members basically work in isolation in daily teaching. | [ ] | [ ] | [ ] | [ ] | [ ] |
| ET7 | Our organization has good flexibility in daily teaching-research activities and performance task allocation. | [ ] | [ ] | [ ] | [ ] | [ ] |
| ET8 | Our organization can adjust its original working methods at any time according to industrial technology iterations and curriculum reform needs. | [ ] | [ ] | [ ] | [ ] | [ ] |
| ET9 | In our organization, the use of digital teaching platforms and information-based teaching resources has become very common. | [ ] | [ ] | [ ] | [ ] | [ ] |
| ET10 | Our organization pays great attention to transforming personalized teaching outcomes and typical practices into shared team resources. | [ ] | [ ] | [ ] | [ ] | [ ] |
| ET11 | While pursuing school running performance, our organization can provide space for teachers’ sustainable professional development. | [ ] | [ ] | [ ] | [ ] | [ ] |
| ET12 | Overall, our organization still maintains a traditional closed management model, with almost no signs of transition toward an open ecology. | [ ] | [ ] | [ ] | [ ] | [ ] |

**2. Primary-level Teaching Organization Leadership Scale (GL)**

| Code | Items | Strongly disagree (1) | Disagree (2) | Neutral/Uncertain (3) | Agree (4) | Strongly agree (5) |
| --- | --- | --- | --- | --- | --- | --- |
| GL1 | The organization leader can clearly express periodic development priorities and goals to the team. | [ ] | [ ] | [ ] | [ ] | [ ] |
| GL2 | The organization leader can quickly translate the school’s administrative planning into specific work goals for teachers. | [ ] | [ ] | [ ] | [ ] | [ ] |
| GL3 | The organization leader respects teachers’ own professional judgments when arranging daily teaching-research or task division. | [ ] | [ ] | [ ] | [ ] | [ ] |
| GL4 | The organization leader has a democratic personality and is very willing to listen to different work opinions proposed by teachers. | [ ] | [ ] | [ ] | [ ] | [ ] |
| GL5 | For teaching explorations initiated by teachers, the organization leader can provide practical flexibility and care. | [ ] | [ ] | [ ] | [ ] | [ ] |
| GL6 | The organization leader encourages and institutionally allows members to try new school-enterprise collaborations or working methods. | [ ] | [ ] | [ ] | [ ] | [ ] |
| GL7 | When facing major construction tasks, the leader usually only issues task indicators and rarely coordinates external resources to provide support. | [ ] | [ ] | [ ] | [ ] | [ ] |
| GL8 | The organization leader is open, fair, and just in task allocation, evaluation, and outcome feedback. | [ ] | [ ] | [ ] | [ ] | [ ] |
| GL9 | The organization leader can promptly recognize teachers’ hard work and unique contributions in public or private ways. | [ ] | [ ] | [ ] | [ ] | [ ] |
| GL10 | When teachers propose improvement suggestions that conflict with current routine management, the organization leader usually tends to maintain the status quo. | [ ] | [ ] | [ ] | [ ] | [ ] |

**3. Teacher Collaboration Scale (TC)**

| Code | Items | Strongly disagree (1) | Disagree (2) | Neutral/Uncertain (3) | Agree (4) | Strongly agree (5) |
| --- | --- | --- | --- | --- | --- | --- |
| TC1 | Teachers in this organization frequently share (e.g., multiple times a month) their self-made course materials and courseware resources with each other. | [ ] | [ ] | [ ] | [ ] | [ ] |
| TC2 | Teachers in this organization exchange information on student learning, teaching difficulties, and classroom problems. | [ ] | [ ] | [ ] | [ ] | [ ] |
| TC3 | Teachers in this organization can conduct practical discussions on the rationality of professional curriculum standards and talent cultivation programs. | [ ] | [ ] | [ ] | [ ] | [ ] |
| TC4 | Teachers in this organization regularly and jointly design the teaching content or practical project tasks of a certain course. | [ ] | [ ] | [ ] | [ ] | [ ] |
| TC5 | Teachers in this organization mainly work independently during lesson preparation and teaching, and rarely conduct collective lesson presentation. | [ ] | [ ] | [ ] | [ ] | [ ] |
| TC6 | Teachers in this organization conduct in-depth problem analysis of multi-dimensional evaluation results, such as end-of-term student evaluations and student grades. | [ ] | [ ] | [ ] | [ ] | [ ] |
| TC7 | Teachers in this organization jointly apply for and deeply participate in research projects, quality course construction, or teaching competitions. | [ ] | [ ] | [ ] | [ ] | [ ] |
| TC8 | Our organization has many routine teaching-research administrative meetings, but they rarely involve professional analysis to resolve actual teaching confusion. | [ ] | [ ] | [ ] | [ ] | [ ] |
| TC9 | Teachers in this organization can conduct high-frequency collaborative teaching and seminars across specialties or broad course categories. | [ ] | [ ] | [ ] | [ ] | [ ] |
| TC10 | Teachers in this organization can carry out deep teaching development collaboration with part-time teachers from the industry or corporate mentor teams. | [ ] | [ ] | [ ] | [ ] | [ ] |
| TC11 | When teachers encounter obstacles in scientific research, professional title evaluation, or teaching, they can obtain interpersonal help from colleagues without psychological burden. | [ ] | [ ] | [ ] | [ ] | [ ] |
| TC12 | Even if there are disagreements among teachers in this organization regarding teaching-research reforms, they can reach a consensus through constructive debate. | [ ] | [ ] | [ ] | [ ] | [ ] |

**4. Perceived Organizational Adaptability Scale (OA)**

| Code | Items | Strongly disagree (1) | Disagree (2) | Neutral/Uncertain (3) | Agree (4) | Strongly agree (5) |
| --- | --- | --- | --- | --- | --- | --- |
| OA1 | Our organization can sensitively receive the latest guidelines on vocational education and quickly fine-tune relevant teaching-research directions. | [ ] | [ ] | [ ] | [ ] | [ ] |
| OA2 | Our organization can promptly iterate teaching content based on the most cutting-edge industrial technological changes and job requirements. | [ ] | [ ] | [ ] | [ ] | [ ] |
| OA3 | Guided by student evaluations or feedback, our organization can quickly reorganize or streamline some outdated teaching methods. | [ ] | [ ] | [ ] | [ ] | [ ] |
| OA4 | When the school makes directional adjustments to the syllabus due to external development strategies, our organization is slow to adjust functional divisions. | [ ] | [ ] | [ ] | [ ] | [ ] |
| OA5 | Our organization can regard the cooperation feedback from external enterprises as a key input for the team to revise practical training plans. | [ ] | [ ] | [ ] | [ ] | [ ] |
| OA6 | When sudden or emergency work temporarily increases, our organization still has mature backup plans to maintain normal teaching standards. | [ ] | [ ] | [ ] | [ ] | [ ] |
| OA7 | Our organization can do its utmost to provide training and off-campus academic visit opportunities for teachers adapting to new courses, new technologies, and new businesses. | [ ] | [ ] | [ ] | [ ] | [ ] |
| OA8 | Our organization has demonstrated strong receptivity in adapting to the integrated transformation of digital teaching (e.g., virtual simulation, digital large screens). | [ ] | [ ] | [ ] | [ ] | [ ] |
| OA9 | Our organization has open and safe feedback channels, allowing teachers to truthfully express their exhaustion and demands for resource allocation when adapting to reforms. | [ ] | [ ] | [ ] | [ ] | [ ] |
| OA10 | Facing the rapidly changing external technological revolution, our organization is accustomed to maintaining old operating routines and has a low willingness for self-iteration. | [ ] | [ ] | [ ] | [ ] | [ ] |

**S1.6 Attention Control Screening Item (Attention Check)**

At a random position in the questionnaire, an auxiliary item was inserted to screen for aimless random responses or machine voting:

Internal variable code: Q1_attention_check

Item expression: “For the accuracy of systematic analysis, please select ‘Agree’ directly for this question.”

Screening rule: Respondents must select the evaluation grade “4 (Agree)”; samples choosing other values or leaving this row blank will be treated as invalid during the data cleaning stage.
