# Supplementary material for: Shaping teachers' challenge appraisals of organizational transition: the roles of grassroots leadership, peer collaboration, and boundary-spanning experience
Source: Front Psychol. 2026 Oct 7;17:1949606. doi: 10.3389/fpsyg.2026.1949606 (PMC13682965; doi:10.3389/fpsyg.2026.1949606)
Supplement: Supplementary file 2 [file Data_Sheet_2.docx]

Supplementary Material S2: Codebook for Dataset

**Table S2-1: Demographic and Control Variables Codebook**

| Variable Name | Variable Label | Type | Categories / Coding Rule |
| --- | --- | --- | --- |
| respondent_id | Respondent ID | Numeric | Continuous integer setting from 1 to N |
| consent | Informed consent confirmation | String / Categorical | Agree (Agree to participate) / Disagree (Disagree) |
| eligibility | Eligibility filtering | String / Categorical | Eligible and willing to continue (Fully meet qualifications) / Not eligible (Ineligible) |
| gender | Respondent gender | String / Categorical | Female (Female) / Male (Male) / Other or Prefer not to say (Other/Prefer not to say) |
| age | Age stage | String / Categorical | 18 to 30 / 31 to 40 / 41 to 50 / 51 and above / Prefer not to answer |
| education | Highest degree | String / Categorical | Associate degree or below (Junior college and below) / Bachelor degree (Bachelor’s degree) / Master degree (Master’s degree) / Doctoral degree (Doctoral degree) |
| professional_title | Professional title level | String / Categorical | Not rated (Unrated) / Junior (Primary) / Intermediate (Intermediate) / Associate senior (Associate senior) / Senior (Senior) |
| college_work_years | Total years of teaching in vocational colleges | String / Categorical | 3 years or less / 4 to 10 years / 11 to 20 years / 21 years or more |
| current_org_years | Years of service in the current primary-level organization | String / Categorical | Less than 1 year / 1 to 3 years / 4 to 6 years / 7 to 10 years / More than 10 years |
| role | Current primary identity/position | String / Categorical | Full-time teacher (Full-time teacher with no administrative position) / Part-time teacher (Part-time teacher) / Teaching office director or course group leader (Teaching-research section director/Course group leader) / Program leader or professional cluster leader (Specialty leader/Professional group leader) / Related institutional administration, etc. |
| organization\_type | Primary-level teaching organization type | String / Categorical | Teaching office (Teaching and research section) / Course group (Course group) / Professional teaching team (Professional teaching team) / Professional cluster teaching organization (Professional group teaching organization) / Teaching innovation team (Teacher teaching innovation team) / Industry-education integrated teaching team (Industry-education integration teaching team) / Other (Other). |
| organization_size | Total scale of personnel covered by the primary-level organization | String / Categorical | 5 or fewer / 6 to 10 / 11 to 20 / 21 to 30 / 31 or more |
| teaching_field | Main specialty/course categories covered | String / Categorical | Electronic information (Electronic information class) / Finance and commerce (Finance and trade class) / Equipment manufacturing (Equipment manufacturing class), etc., 12 standard catalog categories |
| dual_teacher_or_industry_experience | Double-qualified/industry experience characteristics | String / Categorical | Yes certified dual-qualified teacher (Yes, officially certified as a “double-qualified” teacher) / Yes substantial industry practice experience (Yes, not certified but with rich industry experience) / No (No) / Not sure (Uncertain) |

**Table S2-2: Measurement Items Codebook**

(Scoring criteria are all based on a typical 5-point Likert scale: 1 = Strongly disagree, 2 = Disagree, 3 = Neutral/Uncertain, 4 = Agree, 5 = Strongly agree.)

| Construct | Item Name in Data | Item Academic Description | Reverse Coding Rule |
| --- | --- | --- | --- |
| ET | ET1 ~ ET12 | Identifies the “Cognitive Appraisals of Transition Scale” (Ecological Transition) (contains 12 continuous measurement sub-items). | Items ET6 and ET12 in the test items are [reverse-scored items] in the raw data table. A reverse transformation of 6 - x must be performed before analysis. |
| GL | GL1 ~ GL10 | Identifies the “Primary-level Teaching Organization Leadership Scale” (Leadership) (contains 10 continuous measurement sub-items). | Items GL7 and GL10 in the test items are reverse-scored items and need to be reversed before analysis. |
| TC | TC1 ~ TC12 | Identifies the “Teacher Collaboration Scale” (Teacher Collaboration) (contains 12 continuous measurement sub-items). | Items TC5 and TC8 in the test items are reverse-scored items and need to be reversed before analysis. |
| OA | OA1 ~ OA10 | Identifies the “Perceived Organizational Adaptability Scale” (Organization Adaptability) (contains 10 continuous measurement sub-items). | Items OA4 and OA10 in the test items are reverse-scored items and must be reversed before analysis. |
| Q1 | Q1_attention_check | Random funnel variable: Attention control screening red line point (Attention Check). “For the accuracy of systematic analysis, please select ‘Agree’ directly.” | The value in the valid data column must be strictly 4. Any records that are not this value or are system missing values, their corresponding observation rows cannot be included in the formal observation sample. |
